# Predictors of Willingness to Receive Monkeypox Vaccine in Palestine: A Cross-Sectional Study

**DOI:** 10.3390/vaccines13121205

**Published:** 2025-11-29

**Authors:** Nuha El Sharif, Muna Ahmead, Munera Al Abed

**Affiliations:** 1 Faculty of Public Health, Al Quds University, Jerusalem 51000, Palestine; munaahmead@yahoo.com; 2 United Nations Relief and Works Agency for Palestine Refugees in the Near East, Amman 11814, Jordan; mu.alabed@unrwa.org

**Keywords:** monkeypox, vaccine, trust, perception, willingness

## Abstract

**Background/Objective**: While no human monkeypox (MPXV) infections have been reported in Palestine, the rapid global increase in cases, including in neighboring countries, necessitates proactive public health preparedness. This study aimed to assess Palestinians’ willingness to receive MPXV vaccination and to identify associated predictors in the context of a potential outbreak. **Methods**: A cross-sectional online survey was conducted in September 2024. The questionnaire gathered data on participants’ sociodemographic characteristics, risk perceptions, Vaccine Trust Indicator (VTI) scores, vaccination history, and willingness to receive an MPXV vaccine. Bivariate analyses were performed using Pearson’s chi-square test, and a multivariate logistic regression model was employed to identify the determinants of MPXV vaccination willingness. **Results**: The overall willingness to receive MPXV vaccination was low (28.8%). Key findings included significant public misconceptions and concerns: 33% of respondents believed that natural immunity from infection was sufficient, while 43% expressed concerns about potential adverse effects, similar to those associated with COVID-19 vaccines. Furthermore, nearly 60% of participants stated they would decline a free MPXV vaccine. Multivariate analysis revealed that prior COVID-19 vaccination (aOR = 3.07, *p* < 0.05), a moderate VTI score (aOR = 6.65, *p* < 0.05), and prior influenza vaccination (aOR = 4.00, *p* < 0.05) were significant predictors of MPXV vaccination willingness. Willingness to pay for the vaccine also positively influenced vaccination intent. One of the common misconceptions found was the belief that having received a smallpox vaccination prior reduces the need for an MPXV vaccination. **Conclusions**: The willingness to receive an MPXV vaccine in Palestine is suboptimal. Prior vaccination behaviors and general trust in vaccines are key determinants of acceptance. These findings underscore the critical need for public health strategies focused on strengthening trust in vaccine efficacy and safety, along with targeted health education to enhance community preparedness for a potential MPXV outbreak.

## 1. Introduction

Monkeypox (MPXV) is an emerging zoonotic disease caused by the monkeypox virus, an orthopoxvirus in the Poxviridae family [1]. In 2022, the World Health Organisation declared the global outbreak of MPXV a Public Health Emergency of International Concern [2]. Despite the past challenges posed by the coronavirus disease (COVID-19) pandemic, the recent emergence of MPXV has raised concerns among public health authorities regarding its potential as a significant new threat [1,3]. In the context of emerging epidemics, governments should demonstrate caution in the decision-making processes regarding health policies. These implemented policies are often influenced by the public’s responses, which are, in part, dependent upon individual perceptions of the disease and the capacity to modify behaviors in response to evolving circumstances [4]. Currently, there is no specific antiviral treatment approved for MPXV, recommended management relies on standard protocols, including supportive care, symptomatic treatment, and the management of secondary bacterial infections [5,6]. Consequently, public preventative behaviours are essential and effective in preventing the transmission of monkeypox and managing its spread. Consequently, there is significant concern about the population’s vaccination status against monkeypox.

Vaccines are one of the greatest tools available to combat infectious diseases. Thus, it is vital to investigate the population’s willingness to vaccinate and pay for this vaccine [7], as this willingness is strongly influenced by levels of vaccine trust [8]. Vaccine trust reflects people’s attitudes towards vaccination. It is characterized by individual-level confidence in the efficacy and safety of vaccines, as well as in the healthcare personnel and systems providing them. In addition, it is an important factor in vaccine decision-making and acceptance [8]. The Vaccine Indicator Trust (VTT) is a scale that quickly evaluates overall vaccine confidence in individuals or populations, tracks changes over time, or measures the effectiveness of interventions to enhance adult vaccine confidence [9,10,11]. This scale was validated in six countries (the US, UK, Mexico, France, and China), where a strong association was found between the vaccine trust score and intent to receive the influenza vaccine [11].

Monkeypox has been documented in several countries neighbouring Palestine [12,13,14]. Although the MPXV vaccines are currently not available in Palestine, the Palestinian Authority is enhancing its surveillance for early detection of MPXV cases, along with capacity building of healthcare professionals on proper identification of cases. Investigating the determinants associated with vaccine willingness can inform future vaccination promotion strategies [15]. In addition, examining vaccine willingness and its underlying factors might facilitate the development of informed interventions and educational strategies to enhance vaccination rates, ultimately leading to effective control of infectious disease transmission via population immunity [16,17,18]. We previously demonstrated that the Palestinian population exhibited a low level of knowledge about MPXV infection, its mode of transmission, and the incubation period [19]. Currently, there is scant evidence regarding the public’s perceptions of the risks associated with MPXV in Palestine. Furthermore, there is insufficient data regarding willingness to vaccinate against MPXV and the potential impact of the COVID-19 pandemic on those intentions. This study investigates Palestinian willingness to vaccinate against MPXV and how they are associated with risk perception and vaccine trust in the context of a potential pandemic. Our findings can inform communication campaigns and behavioural interventions.

## 2. Materials and Methods

### 2.1. Study Design and Sampling

A descriptive cross-sectional survey was conducted in September 2024. All West Bank Palestinian adults aged 18 and older were targeted. Based on a 95% confidence interval and a 3% margin of error, 50% of respondents would have enough MPXV knowledge; hence, the study chose a sample size of 1067 [20]. Data were gathered using an anonymous, online, self-administered survey. Enketo Express for KoboToolbox was used to generate an electronic questionnaire. Participants were recruited using the snowball sampling technique through personal contacts and social media platforms, including WhatsApp and Facebook Messenger. Participants were requested to disseminate the link nationwide, resulting in 1241 responses.

### 2.2. Study Instrument

The questionnaire was divided into four sections. Section 1 includes the demographic characteristics of the participants, including age, gender, marital status, education level, employment status, monthly income, healthcare work, and location of residence.

Section 2 included participants’ risk perceptions of monkeypox. It consisted of 6 composite variable statements, which were answered on a 5-point Likert scale ranging from ‘very poor’ to ‘very good’: ‘*My health will be severely damaged if I contract monkeypox.’ ‘I think novel Monkeypox is more severe than COVID-19*’, *‘Even if I fall ill with another disease*, *I will not go to the hospital because of the risk of getting Monkeypox there’*, *‘Monkeypox will inflict serious damage in my community’*, *‘I am scared of Monkeypox’* and *‘I am very concerned about this outbreak’* [21]. The composite variable for risk perception was calculated as the mean of these questions (scale: 1–5, with ‘very poor,’ ‘poor,’ ‘neutral,’ ‘good,’ and ‘very good’) and was examined as a continuous variable. The internal consistency coefficient (Cronbach’s α) of our study was 0.80.

Section 3 presents the Vaccine Trust Indicator (VTI), which consists of six items addressing different dimensions of vaccine trust [22]. The VTI employs an 11-point Likert scale (0–10), where lower scores reflect “disagreement” with the statement and higher scores denote “agreement.” The score represents an unweighted average of the participant’s responses to each question, scaled to a 100-point system. A Vaccine Trust Indicator (VTI) score below 40 indicates “low trust in vaccines,” a score ranging from 40 to 70 signifies “moderate trust in vaccines,” and a score exceeding 70 represents “high trust in vaccines.” The internal consistency coefficient of our study, Cronbach’s α, was 0.85.

Section 4 includes participants’ knowledge about previous vaccines: “Vaccination against smallpox can be used to prevent MPXV, and ‘People who received the chickenpox vaccine are immunized against MPXV” using a 5-point Likert scale. Also, “In your opinion, is monkeypox a conspiracy or bioterrorism?”, “Do you think that your health authorities are doing enough to safeguard your health from MPXV?”, “Monkeypox can provide Natural immunity preference”, “The vaccine may have a bad effect on health like the COVID-19 vaccine”. There are currently no specific treatments for monkeypox infections. “There is a vaccine that protects against monkeypox”, “ Vaccination against smallpox can be used to prevent MPXV”, “People who received the chickenpox vaccine were immunized against MPXV”. The internal consistency coefficient, Cronbach’s α, was 0.72.

Section 5 included participants’ willingness to receive the vaccine (study dependent variable): “If monkeypox vaccines are available, are you willing to vaccinate?”. Also, participants were asked about their willingness to pay “The highest total price of the monkeypox vaccine you can afford” and “Are you willing to take the vaccine if it is free?” [23].

### 2.3. Data Analysis

All data analysis was performed using IBM SPSS Statistics version 25 (IBM Corporation, Armonk, NY, USA). Descriptive statistics and chi-square tests were calculated to explore variations in VTI overall and study socio-demographic characteristics, participants’ perception score level as a continuous variable, vaccine history, and vaccine willingness. Multivariate logistic regression analysis was employed to identify factors predicting the vaccination willingness (low and moderate levels) of the study participants. The statistical significance was set as *p* < 0.05. Adjusted odds ratio (aOR) and the 95% confidence interval (95% CI) were estimated. All variables in the model were tested for multicollinearity, we checked. The VIF values were below 5 for all variables in the model.

### 2.4. Ethical Approval and Consent to Participate

All research methods adhered to the Helsinki Declaration and received approval from the Al Quds University Research Ethics Committee (Ref: 412/REC/2024). The social media data was acquired and examined in accordance with the platform’s terms of service and all relevant institutional and national regulations. This online survey was administered anonymously. At the beginning of the study, written materials were distributed outlining its objectives and the intended use of data. Participants provided their informed consent by filling out the questionnaire.

## 3. Results

### 3.1. Vaccine Trust Indicator (VTI) Score, Participants’ Vaccination History, and Participants’ Sociodemographic Characteristics

**Figure 1:**
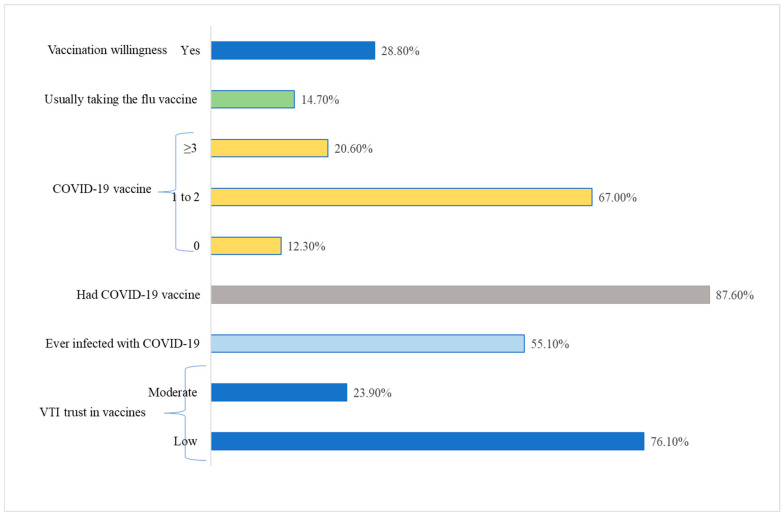
Vaccination willingness, history of vaccination, and Vaccine Trust Indicator (VTI).

The median of VTI was 30 (average 30.05 and SD 13.67). Figure 1 illustrates that, out of 1241 participants, 76.1% (n = 944) exhibit low trust in the vaccine. Furthermore, 55.1% (n = 678) had contracted the COVID-19 virus, while 87.6% had received the COVID-19 vaccine, predominantly 1–2 doses (n = 832). Nevertheless, only 14.7% (n = 192) typically receive the annual influenza vaccination.

Table 1 indicates that almost 50% of the study population was under 35 years of age, 71.4% were female, 66% were not single, and 79.2% had more than 12 years of schooling. A total of 567 participants (46.1%) were from the southern region of the West Bank, 62.4% were employed, with 28% working in the healthcare sector. About 50% of the participants reported a monthly income below 1080 US dollars, and 25% had chronic illnesses (Table 1).

The chi-square (χ2) test was applied to assess the difference in the distribution of vaccination willingness and participants’ socio-demographic characteristics. Because multiple chi-square comparisons were conducted in Table 1, we applied Holm–Bonferroni correction to adjust *p*-values. Results show that participants’ gender, place, area of residence, and working in the health sector were statistically significantly associated with vaccine trust (*p* < 0.05) (See Table 1).

### 3.2. Participants’ Knowledge About Previous Vaccines and Participants’ Willingness to Pay for the Monkeypox Vaccine

Approximately 27% of participants recognized that the smallpox vaccination is ineffective in preventing MPXV infection, whereas about 38% knew that immunization against chickenpox does not protect from MPXV (see Table 2).

The willingness to receive MPXV vaccination was relatively low (28.8%); 33% believe infection can induce natural immunity, while 43% are concerned that it may negatively impact their health, similar to the COVID-19 vaccine. Approximately 60% indicated they would refuse the MPXV vaccination, even if offered at no cost, while 68.5% expressed an unwillingness to pay for it (see Table 2).

### 3.3. Risk Perception of Study Participants Towards Monkeypox

**Figure 2:**
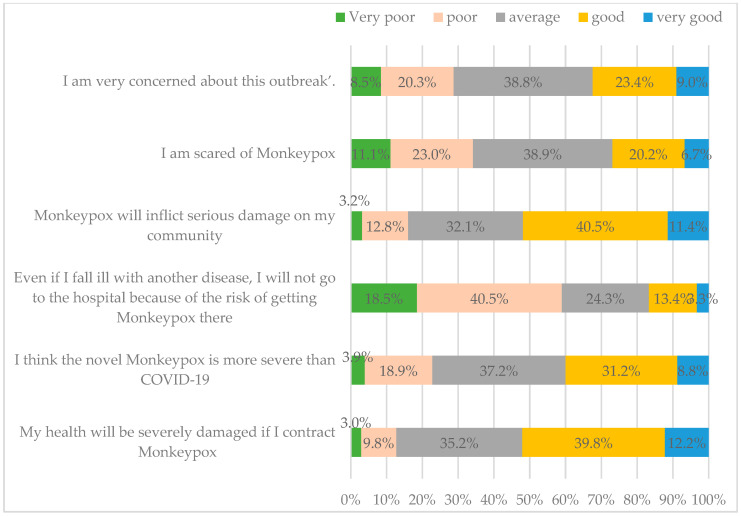
Risk Perception of study participants towards MPXV.

The mean risk perception is 18.5 (standard deviation 4.32), with a median of 18 and a range of 30. Approximately 32.4% of study participants expressed concern regarding the monkeypox outbreak, 26% feared contracting the virus, and 51.9% perceived it as potentially detrimental to the community. Also, 16.7% indicated they planned to avoid seeking hospital care in the case of an outbreak, 40% believed this would not be more severe than the COVID-19 pandemic, and 52.1% anticipated it would impact their health (see Figure 2).

### 3.4. Associations Between Participants’ Risk Perception Score, Vaccination History, Willingness to Get Vaccinated, and MPXV Vaccination Willingness

A statistically significant association was found between the vaccine trust indicator (VTI) and the willingness to vaccinate (*p* < 0.05). Among individuals intending to receive the MPXV vaccine, 52.8% demonstrate a moderate level of trust, in contrast to 12% of those who intend to get vaccinated (Table 3). Also, Table 3 shows that beliefs about the origins of monkeypox showed a significant association with participants’ willingness to vaccinate (*p* = 0.046). Individuals with a neutral perspective on the notion of MPXV as a conspiracy or bioterrorism demonstrated a higher intent to vaccinate. Also, a significant association was seen between trust in local health authorities and vaccination willingness (*p* < 0.001). A higher percentage of individuals planning to vaccinate perceived that health authorities were actively working to protect public health, “maybe”, compared to those who do not intend to get vaccinated. However, satisfaction with social media coverage of the monkeypox outbreak was not significantly associated with vaccination willingness (*p* = 0.084).

The participants’ perception that smallpox and chickenpox protect against monkeypox was strongly associated with receiving the MPXV immunization (*p* < 0.05). Furthermore, they believed that natural immunity or vaccination might provide immunity against infection, which substantially influenced their willingness to receive the vaccine (*p* < 0.05). Conversely, their view that the MPXV vaccine adversely affects health, similar to the COVID-19 vaccine, did not influence willingness to vaccinate (*p* > 0.05) (Table 3).

The participants’ readiness to receive vaccination is significantly correlated with their willingness to vaccinate, with the majority of those intending to get vaccinated expressing a willingness to pay for the vaccine, in contrast to 19.2% of those lacking such willingness (*p* < 0.05). Furthermore, 27% of individuals vaccinated against COVID-19 express a willingness to receive the MPXV vaccination, in contrast to 57.5% of those lacking intent to pay (*p* < 0.05). However, the difference was not statistically significant among those infected with the COVID-19 virus (*p* > 0.05). Moreover, individuals who typically receive the influenza vaccine exhibited a heightened willingness to obtain the MPXV vaccine compared to others (Table 3). Finally, there was no significant difference between the risk perception and willingness to get the MPXV vaccine.

### 3.5. Multivariate Analysis for the Determinants of Willingness to Get Vaccinated for MPXV

Our analysis revealed that being male significantly increases the likelihood of intending to get MPXV vaccination compared to being female (aOR 1.57, *p* < 0.05). Furthermore, individuals with 12 years or less of education showed a greater willingness to receive vaccination compared to those with more than 12 years of education (aOR 1.71, *p* < 0.05). Notably, the current COVID-19 immunization status was a significant predictor (adjusted Odds Ratio (aOR) 3.07, 95% CI 1.18–2.47), as illustrated in Table 4. Comparably, a moderate score on the Vaccine Trust Indicator (VTI) was associated with increased likelihood of accepting the Monkeypox vaccine (aOR 6.65, 95% CI 4.77–9.28), and individuals who typically received the influenza vaccine were four times more likely to accept the Monkeypox vaccine as well. Moreover, the ability to cover the expense of the MPXV vaccine was a significant determinant of vaccination uptake. An incorrect assumption that the smallpox vaccine protects against MPXV substantially increases the willingness to receive vaccination by two to three times in comparison to others.

Multivariate logistic regression includes: Previously infected with COVID-19; had COVID-19; number of COVID-19 vaccines; and do you usually take the influenza vaccine? If MPXV vaccines are available, are you willing to vaccinate? MPXV can have a natural immunity preference; the vaccine may have a bad effect on health like the COVID-19 vaccine; are you willing to take the MPXV vaccine if it is free? The highest total price of the MPXV vaccine you can afford is what; my health will be severely damaged if I contract MPXV; I think the novel MPXV is more severe than COVID-19; Even if I fall ill with another disease, I will not go to the hospital because of the risk of getting MPXV there; MPXV will inflict serious damage on my community; I am scared of MPXV; I am very concerned about this outbreak; there are currently no specific treatments for MPXV infections; there is a vaccine that protects against MPXV after controlling for participants’ age, gender, marital, education level, place of residence, working, work in the health sector, and income level.

## 4. Discussion

Immunization represents a highly efficient health intervention for addressing vaccine-preventable diseases. Globally, vaccine hesitancy is recognized as a significant challenge to the successful implementation and acceptance of vaccination programs [24]. This study in Palestine is the first to examine the risk perception and trust associated with vaccination willingness during a new potential epidemic.

### 4.1. Willingness to Receive the MPXV Vaccination

Our findings showed that the willingness to receive the MPXV vaccination was low (28.8%). A systematic review showed that the worldwide willingness to vaccinate against MPXV was 61%, with African countries showing a rate of 43% and European countries at 62%. In the Arab countries, the willingness to vaccinate against MPXV was as follows: 29% among Jordanians, 57% among Lebanese, 39% among Algerians, 26% among Iraqis, and 47% among Saudis [25]. Therefore, the Palestinian willingness to vaccinate is somewhat lower than in several countries, but similar to Iraq and Jordan. In the prevent study, two-thirds of the Vietnamese population expressed a willingness to receive the vaccination; nevertheless, vaccine hesitancy was predominantly attributed to a lack of awareness regarding MPXV and the vaccine itself [26]. This disparity among nations may arise from differing responses to the severity of an illness and the implementation of preventive measures, driven by socioeconomic and cultural factors, information accessibility, and distrust in government and healthcare systems. In Palestine, public trust in health authorities is often low due to fragmented governance, lack of resources, fragmented healthcare infrastructure across different administrative zones, and inconsistent communication. Additionally, cultural beliefs, including community-based health information sharing and traditional health beliefs, and limited access to accurate health information contribute to varied perceptions of disease severity and vaccine acceptance.

Another finding of this study is that 33% believed that the MPXV infection could induce natural immunity, whereas 43% were concerned about its negative impact on their health. Approximately 60% indicated that they would decline the MPXV immunization, even if provided at no cost, while 68.5% expressed reluctance to purchase it. Similar findings exhibited variability across studies conducted in countries with a higher prevalence of MPXV compared to those in nations with lower prevalence [14,27,28]. In Jordan, where the incidence of the MPXV virus is low, nurses and physicians had a low willingness (28.9%) to get the MPXV vaccination. The psychological factors and prior vaccination behavior emerged as the most significant determinants of MPXV vaccine acceptance and attitudes towards mandatory vaccination [29]. In Vietnam, participants’ readiness to take and pay for the monkeypox vaccine was influenced by their understanding of transmission and infection risk, whereas financial constraints and vaccine apprehension were significant factors contributing to hesitation [26]. Past studies on the COVID-19 vaccine hesitancy in Palestine have shed light on certain psychological factors and vaccination practices that are likely to be present in other new vaccines as well [30,31]. These include an overall resistance to new immunizations, doubts about the safety and effectiveness of vaccines, worries about unanticipated side effects, and a preference for natural immunity.

### 4.2. Vaccination History and Willingness to MPXV Vaccination

Our study findings revealed that COVID-19 vaccination status significantly predicted the willingness to receive the MPXV vaccine. Several studies have indicated a significant association between COVID-19 vaccination status and the willingness to receive monkeypox vaccination [21,29,32]. This relationship may serve as a reflection of how the pandemic experience has shaped public attitudes towards the monkeypox outbreak. Further studies on MPXV vaccine acceptance have identified similar factors of vaccine acceptance, such as perceived risk, vaccine efficacy, private experiences with COVID-19, and vaccination experience [28,33,34,35,36]. This may indicate that even in the absence of mandatory vaccination, it would be valuable to investigate the participants’ willingness to accept the MPXV vaccine, as this would offer insight into their likely responses during future outbreaks.

Our study also showed that having the influenza vaccine was a strong predictor of willingness to be vaccinated against MPXV. The vaccination history of an individual can impact their willingness to receive vaccines, as prior immunity is likely to influence their acceptance of further vaccinations [29,37]. In China, the history of influenza vaccination was associated with women’s acceptance of the influenza vaccine during the COVID-19 pandemic [38].

Research indicates that those who have received regular vaccinations, such as the influenza vaccine, are more willing to accept new immunizations, including the monkeypox vaccine. This is due to their previous vaccination history.

In our study, it was observed that participants who had received the smallpox vaccine demonstrated a greater willingness to accept vaccination against Monkeypox. Some studies indicate that the smallpox vaccine demonstrates an effectiveness ranging from 35% to 85% in preventing MPXV, accompanied by a low incidence of adverse events [39,40,41]. A systematic review and meta-analysis have demonstrated that smallpox immunization significantly decreases the probability of MPXV infection and severe disease, offering substantial proof for its clinical importance in monkeypox prevention [40]. Consequently, additional study is required to assess the degree to which various smallpox vaccines provide cross-protection against MPXV, and the creation of specialized vaccines for MPXV is imperative. In summary, previous experience with vaccination, like the COVID-19 and the influenza vaccine in Palestine, emphasizes the necessity to customize comprehensive communication strategies for individuals lacking a flu vaccination history, tackling vaccine hesitancy, distrust in authorities, and misinformation, while enhancing vaccine infrastructure and health literacy to render future vaccination campaigns more inclusive.

### 4.3. Vaccine Indicator Trust (VIT) and Willingness to Vaccinate

Our study, Vaccine Indicator Trust (VTI), demonstrated a significant association with the MPXV immunization. Participants had either low (less than 4 out of 10) or medium (between 4 and 7) scores, but none of the participants had a high score (>7) on VTI. Comparable results were observed in a population-based study conducted in the United States. A high score on the VTI has been associated with a greater likelihood of accepting the MPXV vaccine (AOR 5.4, 95% CI 3.2–0.1) compared to those with medium VTI scores. Individuals with poor scores were markedly hesitant to accept a suggested MPXV immunization [21]. The study highlighted the effectiveness of the VIT scale. The identification of vaccination as a significant predictor within the context of a healthy lifestyle suggests a potentially effective approach to framing vaccination initiatives. A study conducted in Saudi Arabia revealed that trust in the Ministry of Health (57.7%) and the perception of the vaccine as a social responsibility (44.6%) were the primary factors influencing healthcare professionals’ willingness to get the monkeypox vaccine [42]. In Yemen, 57.7% of the study participants feared human MPXV, and 47.7% said it was a conspiracy that undermines trust and may promote vaccine hesitancy [43]. Our study participants who are unwilling to receive the vaccine perceive the monkeypox virus as a plot or an act of bioterrorism. But those who intend to take the vaccine perceive that health authorities are sufficiently protecting their health from MPXV. Similar findings were seen in Kuwait [44], Jordan [45], and Lebanon [14]. Therefore, in the Palestinian context, there should be an education program that targets vaccine hesitancy, mistrust in authorities, and misinformation, which can strengthen the overall health literacy to foster a more receptive population for all future vaccination campaigns.

### 4.4. Risk Perception and Willingness to Vaccinate

Several studies have shown that risk perception has a strong positive association with vaccination willingness [46,47]. While our study investigated the risk perception of the disease, the perceived risk of side effects, and willingness to vaccinate, we could not confirm this association. This could be explained by the study participants’ conflicting ideas, such as recognising the disease as a risk while also fearing vaccination safety, which can undermine a clear directional relationship. Furthermore, external factors such as misinformation, low trust in health authorities, or social norms may have had a greater impact on vaccination willingness than individual risk evaluations alone.

### 4.5. Socio-Demographic Factors and Willingness to Vaccinate

The sociodemographic status of the study participants influences their willingness to receive the MPXV vaccine. Males in our study demonstrated a higher likelihood of vaccine acceptance than females. Similar findings demonstrate variability across studies [23,28,29]. In Ghana, females demonstrated a significantly higher willingness to accept the MPXV vaccine (aOR 1.65; 95% CI: 1.13–2.40) compared to males [37]. A systematic review revealed that the pooled odds ratio (OR) from studies conducted in China, the USA, Peru, France, Belgium, and the Netherlands demonstrated that females had a lower willingness to receive the vaccine compared to males, with an OR of 0.61 (95% CI, 0.43–0.86) [32]. The justification for these findings is that older women and pregnant women exhibit higher hesitancy for vaccination, as demonstrated in many studies [32]. This may reflect the expected influence of potential side effects and unverified information on social media, which could be explained by women’s complex familial responsibilities and roles.

Furthermore, individuals with 12 years or less of education showed a greater willingness to receive vaccination compared to those with more than 12 years of education (aOR 1.71, *p* < 0.05). Several studies found that improving acceptance of monkeypox vaccination involves several factors, including education, communication, community engagement, and trust-building. In Saudi Arabia, a higher education level was associated with low agreement with vaccination [48]. Also, in China, higher monkeypox knowledge was related to a higher willingness to vaccinate [23]. Consequently, these findings indicate that a comprehensive initiative to spread awareness regarding MPXV is imperative for individuals with lower educational attainment in Palestine. Recently, various educational initiatives concerning MPXV infection have been disseminated through local radio stations; nonetheless, this endeavor is considered inadequate.

### 4.6. Strengths & Limitations

The strength of our study is attributed to the usage of a representative sample of the adult Palestinian population, which allowed for a rapid assessment of vaccination attitudes, knowledge gaps, and communication needs in the event of a potential outbreak. However, the findings should be interpreted with caution due to several limitations. As a cross-sectional study, it provides correlational findings but cannot establish causal relationships. The online survey format and use of snowball sampling likely introduced selection bias; therefore, our sample’s demographic composition may not precisely reflect the nationwide distribution. Furthermore, those intending to receive COVID-19 vaccinations may differ from the general population at risk for MPXV. As a result, our findings should be interpreted in the context of those who seek medical care. As a result, we should use caution when generalizing our findings.

Furthermore, because the monkeypox epidemic has not yet significantly evolved in Palestine, our findings represent the current situation and serve as a baseline for future research. Despite these limitations, this study provides valuable nationwide cross-sectional data essential for developing vaccination programs related to monkeypox. The absence of MPXV outbreaks in Palestine during the study period may have influenced participants’ knowledge and risk perception of the disease. Nonetheless, our study is more useful because it shows baseline knowledge and risk perception in a setting where the disease is not very common, which is useful for public health preparedness. Identifying knowledge gaps and misunderstandings before an outbreak can inform targeted educational programs and preparedness strategies.

## 5. Conclusions

The study found that Palestinian communities had a low willingness to receive the monkeypox vaccine, with identifiable factors including education level and male gender, alongside prior vaccination experience (like COVID-19, smallpox, or influenza). Critically, the Vaccine Trust Indicator was significantly associated with the willingness to vaccinate. These findings underscore the urgent need for context-specific communication and education strategies to boost vaccination rates. By identifying groups with high refusal rates, targeted initiatives can address concerns and enhance vaccine confidence, ultimately crucial for achieving optimal vaccination coverage and global public health safety.

Current immunization strategies prioritize infectious diseases, but no general population recommendation exists for the monkeypox vaccine. These findings underscore the importance of effective communication strategies to improve vaccination attitudes and willingness among the Palestinian population. Future communication efforts must actively address the growing prevalence of myths and misinformation, highlighting the need for robust approaches to strengthen vaccine acceptance and understanding. Further research should focus on assessing MPXV vaccine acceptance, defined as an individual’s willingness to receive or adopt a vaccine based on their confidence in its effectiveness and perceived safety.

A collaborative strategy involving the Ministry of Health, the community, and social media is vital for the success of vaccination programs. The Ministry of Health is responsible for formulating policies, disseminating accurate information, and implementing campaigns. Communities, through their leaders and networks, play a key role in fostering trust, sharing information, and addressing local concerns. While social media carries the risk of spreading misinformation, it also serves as a powerful platform for engagement, rapid and targeted communication, and myth-busting. A coordinated, multi-channel approach is therefore essential to increasing vaccine uptake and protecting public health.

## Figures and Tables

**Table 1:** Monkeypox vaccination willingness by demographics (n = 1241).

			Monkeypox Vaccination Willingness					
			No/Do Not Know		Yes		χ2	Holm-Corrected *p* Value
		Total	N	%	0.010	%	*p*-Value	
Overall		1241	883	71.2%	358	28.8%		
Age in years	18–25	327	224	25.4%	104	29.1%	0.009	0.063
	26–35	343	238	27.0%	106	29.6%		
	36–50	413	323	36.6%	96	26.8%		
	>51	147	98	11.1%	52	14.5%		
Gender	Male	352	227	25.7%	129	36.0%	<0.001	0.010
	Female	878	656	74.3%	229	64.0%		
Marital Status	Not single	812	593	67.2%	229	64.0%	0.28	0.84
	single	418	290	32.8%	129	36.0%		
Educational level	≤12 years	255	176	19.9%	88	24.6%	0.07	0.35
	>12	975	707	80.1%	270	75.4%		
Place of Residence	City	557	370	41.9%	189	52.8%	<0.001	0.009
	Village	510	397	45.0%	113	31.6%		
	Refugee camp	163	116	13.1%	56	15.6%		
Area of residence	North	259	213	24.1%	46	12.8%	<0.001	0.008
	Middle	404	284	32.2%	120	33.5%		
	South	567	386	43.7%	192	53.6%		
Work now	Yes	767	547	61.9%	225	62.8%	0.76	0.76
	No	463	336	38.1%	133	37.2%		
Work in the health sector	Yes	353	647	73.3%	241	67.3%	0.035	0.21
	No	876	236	26.7%	117	32.7%		
Monthly income	≤540 $	238	171	19.4%	70	19.6%	0.42	0.84
	541–1080 $	359	265	30.0%	100	27.9%		
	1081–1921 $	270	201	22.8%	71	19.8%		
	>1621 $	189	125	14.2%	64	17.9%		
	No income	174	121	13.7%	53	14.8%		
Have chronic diseases	Yes	308	100	27.9%	208	23.6%	0.10	0.40
	No	933	258	72.1%	675	76.4%		

**Table 2:** Knowledge about vaccines and willingness to get vaccinated and pay for the Monkeypox vaccine (n = 1241).

Variables		Frequency	Percentage
Knowledge about vaccines			
Vaccination against smallpox can be used to prevent MPXV	Low	337	27.2%
	Moderate	437	35.2%
	High	467	37.6%
People who received the chickenpox vaccine are immunized against MPXV	Low	471	38.0%
	Moderate	523	42.1%
	High	247	19.9%
If MPXV vaccines are available, Are you willing to be vaccinated?	Yes	358	28.8%
	No/Don’t know	883	71.2%
MPXV can natural immunity preference	Yes	410	33.0%
	No/Don’t know	831	66.9%
The vaccine may have a bad effect on health, like the COVID-19 vaccine	Yes	534	43.0%
	No/Don’t know	707	57.0%
Willingness to Pay			
Willingness to take the MPXV vaccine if it is free	Yes, if it is free	287	23.1%
	Yes, even with payment	215	17.3%
	I do not want it even if it is free	739	59.5%
The highest total price of the MPXV vaccine you can afford is	Nothing	850	68.5%
	15–30 US $	253	20.4%
	Any price	138	11.1%

**Table 3:** Association between Vaccine Trust Indicator (VTI), knowledge about vaccines, vaccination history, willingness to pay, risk perception score, and MPXV vaccination willingness (n = 1241).

		Monkeypox Vaccination Willingness				
		No/Do Not Know		Yes		χ2
		n	%	n	%	*p* Value
		883	71.2%	358	28.8%	
Vaccine Trust Indicator (VTI)	Low	775	87.8%	169	47.2%	<0.001
	Moderate	108	12.2%	189	52.8%	
Knowledge about vaccines						
In your opinion, is monkeypox a conspiracy or bioterrorism?	Do not know	299	31.3%	85	29.7%	0.046
	Neutral	437	45.8%	152	53.1%	
	Agree	219	22.9%	49	17.1%	
Do you think that your health authorities are doing enough to safeguard your health from MPXV?	I do not know	244	25.5%	38	13.3%	<0.001
	No	439	46.0%	131	45.8%	
	Maybe	272	28.5%	117	40.9%	
How satisfied are you with the social media coverage of the Monkeypox Outbreak?	Least satisfied	385	40.3%	107	37.4%	0.084
	Satisfied	141	14.8%	49	17.1%	
	Average	382	40.0%	120	42.0%	
	Very satisfied	26	2.7%	10	3.5%	
Vaccination against smallpox can be used to prevent MPXV	Low	284	32.2%	53	14.8%	<0.001
	Moderate	334	37.8%	103	28.8%	
	High	265	30.0%	202	56.4%	
People who received the chickenpox vaccine were immunized against MPXV.	Low	347	39.3%	124	34.6%	<0.001
	Moderate	386	43.7%	137	38.3%	
	High	150	17.0%	97	27.1%	
There are currently no specific treatments for MPXV infections	False	618	70.0%	238	66.5%	0.23
	True	265	30.0%	120	33.5%	
There is a vaccine that protects against MPXV	False	702	79.5%	234	65.4%	<0.001
	True	181	20.5%	124	34.6%	
MPXV can natural immunity preference	Yes	276	31.3%	134	37.4%	0.03
	No	607	68.7%	224	62.6%	
The vaccine may have a bad effect on health, like the COVID-19 vaccine	Yes	395	44.7%	139	38.8%	0.06
	No	488	55.3%	219	61.2%	
Willingness to Pay						
Are you willing to take the MPXV vaccine	Yes, if it is free	97	11.0%	190	53.1%	<0.001
	Yes, even if it is with payment	72	8.2%	143	39.9%	
	I don’t want, even if it is free	714	80.9%	25	7.0%	
The highest total price of the MPXV vaccine you can afford is	Nothing	676	76.6%	174	48.6%	<0.001
	50–100	125	14.2%	128	35.8%	
	Any price	82	9.3%	56	15.6%	
Participants’ Vaccination History						
Had the COVID-19 vaccine	No	146	16.5%	22	6.1%	<0.001
	Yes	737	83.5%	336	93.9%	
Number of COVID-19 doses	Not vaccinated	135	15.3%	18	5.0%	<0.001
	1–2	588	66.6%	244	68.2%	
	≥3	160	18.1%	96	26.8%	
Do you usually take the influenza vaccine?	Yes	69	7.8%	113	31.6%	<0.001
	No/Don’t know	814	92.2%	245	68.4%	
Ever being infected with COVID-19	No/Don’t know	411	46.5%	145	40.5%	0.059
	Yes	472	53.5%	213	59.5%	
Risk perception scale (mean, SD)	Mean (SD)	955	18.4 (4.33)	286	18.7 (4.28)	0.62

**Table 4:** Multivariate analysis for determinants of Vaccination Willingness among study participants.

	Sig.	aOR	95% CI for aOR	
			Lower	Upper
Gender (Male/Female)	0.009	1.57	1.12	2.19
Educational level (≤12/>12) years	0.005	1.71	1.18	2.47
Did you have the COVID-19 vaccine (Yes/No)	<0.001	3.07	1.77	5.31
Trust indicator index (VIT) (Moderate/Low)	<0.001	6.65	4.77	9.28
Do you usually take the influenza vaccine? (Yes/No)	<0.001	4.42	2.98	6.58
The highest total price of the MPXV vaccine you can afford	<0.001			
Nothing		Reference		
15–30 US$	<0.001	3.40	2.36	4.90
Any price	0.005	1.93	1.21	3.07
Vaccination against smallpox can be used to prevent MPXV.	<0.001			
Low		Reference		
Medium	0.002	1.99	1.28	3.09
High	<0.001	3.60	2.36	5.51

aOR: adjusted Odds Ratio, 95% CI: 95% confidence interval.

## Data Availability

The data presented in this study are available on request from the corresponding author due to university rules of data sharing.

## Abbreviations

The following abbreviations are used in this manuscript:

Monkeypox	MPXV
VTI	Vaccine Trust Indicator
aOR	Adjusted Odds Ratio
95% CI	95% Confidence Interval
